# Case Report: Thoracoscopic treatment of infradiaphragmatic pulmonary sequestration and intrathoracic kidney associated with congenital diaphragmatic hernia

**DOI:** 10.3389/fped.2024.1442347

**Published:** 2024-08-14

**Authors:** Rui Guo, Chunhua Dong, Yunpeng Zhai, Huashan Zhao, Longfei Lv, Shisong Zhang

**Affiliations:** ^1^ Department of Thoracic and Tumor Surgery, Children’s Hospital Affiliated to Shandong University, Jinan, Shandong, China; ^2^ Department of Thoracic and Tumor Surgery, Jinan Children’s Hospital, Jinan, Shandong, China; ^3^ Department of Medical Imaging, Children’s Hospital Affiliated to Shandong University, Jinan, Shandong, China; ^4^ Department of Medical Imaging, Jinan Children’s Hospital, Jinan, Shandong, China

**Keywords:** thoracoscopy, infradiaphragmatic pulmonary sequestration, intrathoracic kidney, congenital diaphragmatic hernia, enhanced computed tomography

## Abstract

**Background:**

Congenital pulmonary sequestration is a rare lung anomaly that can be classified as intralobar pulmonary sequestration or extralobar lung sequestration (ELS). Infradiaphragmatic pulmonary sequestration is a rare type of ELS. Furthermore, intrathoracic kidney (ITK) is a rare disease that can be associated with a congenital diaphragmatic hernia (CHD) in 0.25% of cases. We report the first case of infradiaphragmatic pulmonary sequestration and ITK associated with CDH in a child.

**Case report and management:**

The patient, male, aged 6 months, visited our hospital 2 months prior due to shortness of breath. Based on chest ultrasonography and enhanced computed tomography (CT) examination, infradiaphragmatic pulmonary sequestration and ITK were considered to be associated with CDH. The patient was admitted to our hospital for treatment. After admission, his blood pressure was 85/61 mmHg, there was no hematuria or proteinuria, creatinine was 14 µmol/L, and urea nitrogen was 2.96 mmol/L, all of which showed no abnormalities. A complete preoperative examination was performed prior to surgical treatment. Thoracoscopy revealed that the right kidney had herniated into the chest cavity on the posterolateral side of the diaphragm. The right kidney was returned to the abdominal cavity, the hernia sac was opened, and a bright red lesion tissue with clear boundaries and an abnormal blood vessel supply was observed. After cutting off the abnormal blood vessels, LigaSure TM was used to remove the diseased tissue, and the renal fat sacs and renal tissue were visible. Intermittent suturing of the hernia ring was performed to seal the diaphragmatic hernia. Postoperative pathological examination revealed infradiaphragmatic pulmonary sequestration. The postoperative recovery of the patient was smooth, and a chest CT scan at 2 months showed that the right kidney had returned to the abdominal cavity and the right diaphragm was in the normal position.

**Conclusion:**

Infradiaphragmatic pulmonary sequestration and ITK associated with CDH is extremely rare. A diagnosis and appropriate surgical planning can be developed using enhanced CT. For infradiaphragmatic pulmonary sequestration located at the top of the hernia sac in CHD, thoracoscopic resection of the infradiaphragmatic pulmonary sequestration and repair of the diaphragmatic hernia is feasible and effective.

## 1 Introduction

Congenital pulmonary sequestration (PS) is a rare congenital lung anomaly, accounting for 0.15%–6.4% of all lung deformities (1). PS is classified as intralobar pulmonary sequestration (ILS) and extralobar lung sequestration (ELS), with ELS accounting for 15%–25% of cases of PS (2). ELS is divided into the supradiaphragmatic, intradiaphragmatic, and infradiaphragmatic types, with the lower infradiaphragmatic type being relatively rare (3).

Intrathoracic kidney (ITK) is a rare disease with an incidence of only 1/16,000 (4). Furthermore, ITK associated with congenital diaphragmatic hernia (CHD) is extremely rare, accounting for only 0.25% of cases (5). Currently, there are no reports in the literature of cases of infradiaphragmatic pulmonary sequestration and ITK associated with CDH.

This article reports the case of a child with infradiaphragmatic pulmonary sequestration and ITK associated with CDH who achieved a favorable effect with the use of the thoracoscopic technique for infradiaphragmatic pulmonary sequestration resection and diaphragmatic hernia repair.

## 2 Case presentation

**Figure 1:**
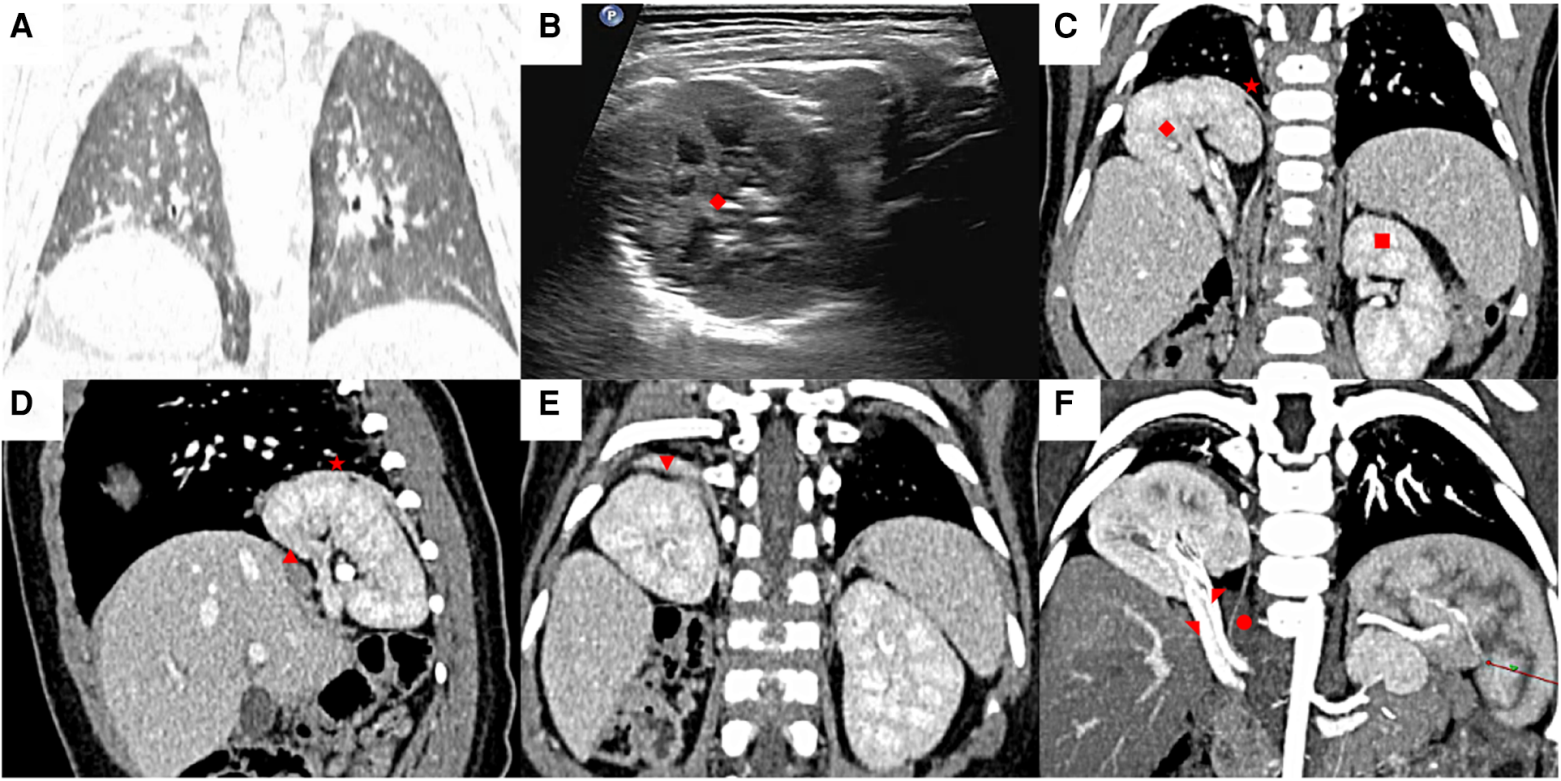
Preoperative ultrasound and chest computed tomography (CT) imaging findings. **(A)** The chest CT coronal image showed right diaphragmatic elevation, suspected an abnormal mass herniated into the chest cavity. **(B)** Chest ultrasound confirmed that the right kidney had herniated into the right chest cavity. **(C)** Contrast enhanced chest CT scan showed that the rotation position of the right kidney was significantly shifted upwards above the diaphragm (coronal position). **(D)** The local discontinuity in the right diaphragm (sagittal position). **(E)** Thickening of the upper wall of the hernia sac with significant enhancement on enhanced scanning. **(F)** The abnormal supply of small blood vessel originating from the right renal artery. (◆: Right kidney, ★: Hernia sac, ▪: Left kidney, ▴: Diaphragmatic muscle, ▾: Infradiaphragmatic pulmonary sequestration, ●: Abnormal blood vessels, ◤: Right kidney artery, ◥: Right kidney vein).

The patient, male, aged 6 months, was admitted to our hospital 2 months prior because of shortness of breath. A chest computed tomography (CT) scan showed an abnormal mass in the right chest cavity, which was suspected to be in the right kidney, with a suggested diagnosis of CDH (Figure 1A). Chest ultrasonography confirmed that the right kidney had herniated into the right chest cavity (Figure 1B). The patient was diagnosed with ITK associated with CDH and was admitted to the hospital for treatment. The patient had no history of pneumonia, cough, sputum production, chest pain, cyanosis of the lips, or breathing difficulties. After admission, a contrast enhanced chest CT scan showed local discontinuity in the right diaphragm, the rotation position of the right kidney was significantly shifted upwards above the diaphragm, and the morphology of the right renal artery and vein was visible without stenosis. A hernia sac shadow could be seen at the discontinuous diaphragm, with thickening and significant enhancement of the upper wall of the hernia sac. Small blood vessels from the renal artery branch were observed running inside, suggesting infradiaphragmatic pulmonary sequestration and ITK associated with CDH (Figures 1C–F).

**Figure 2:**
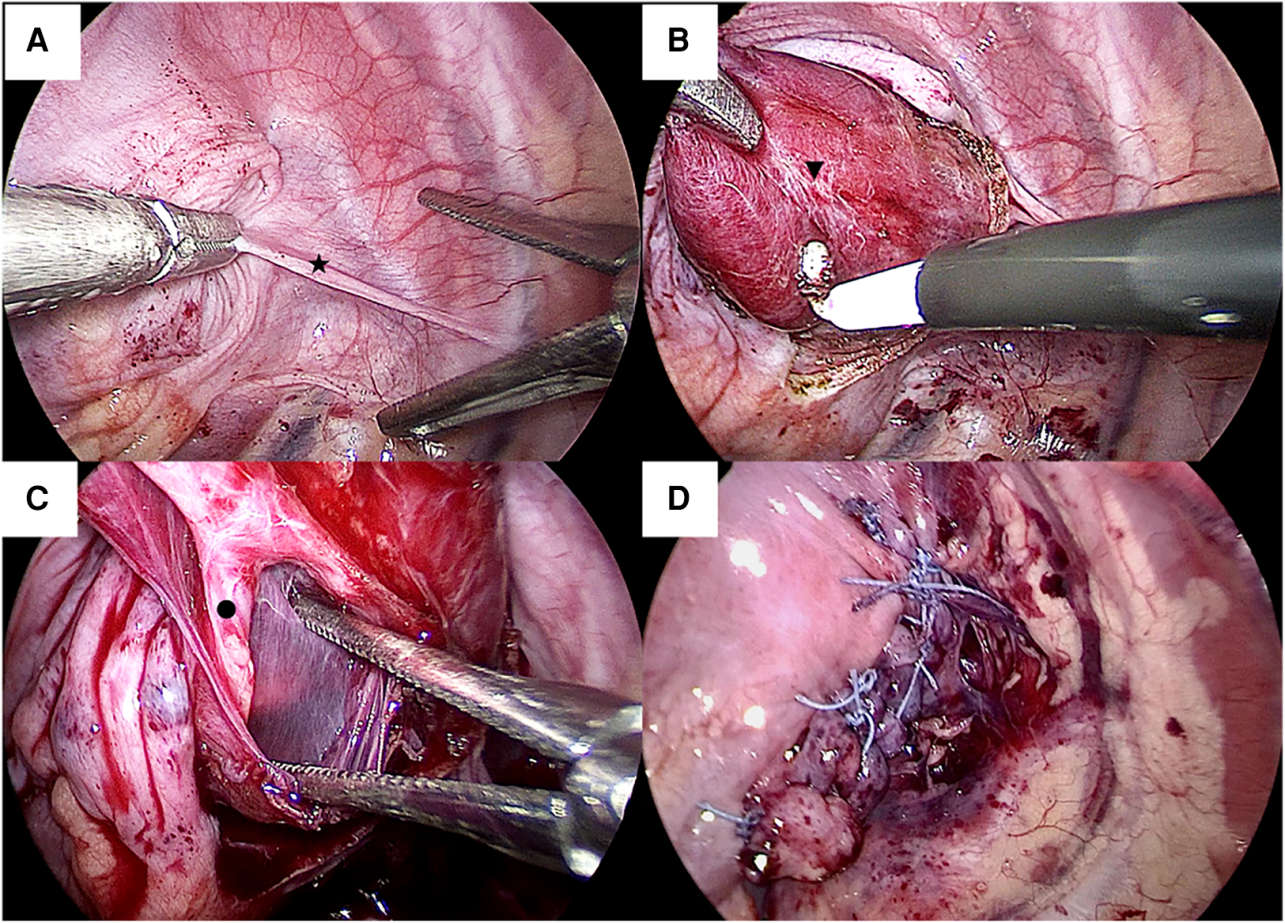
Intrathoracic endoscopic images. **(A)** The right kidney had herniated into the chest cavity on the posterior lateral side of the diaphragm, which was placed back into the abdominal cavity. The hernia sac was thin. **(B)** Upon incision of the hernia sac, a bright red colored lesion with clear boundaries was observed. **(C)** Visible a small abnormal blood vessels supplying the lesion. **(D)** The repair of diaphragmatic hernia was smooth. (★: Hernia sac, ▾: Infradiaphragmatic pulmonary sequestration, ●: Abnormal blood vessels).

The patients’ blood pressure was 85/61 mmHg, there was no instance of hematuria or proteinuria, creatinine was 14 µmol/L, and urea nitrogen was 2.96 mmol/L, which were all within the normal range. A complete preoperative examination was performed prior to surgical treatment. The child laid on the left side with single-lumen tracheal intubation and single-lung ventilation (selective occlusion of the affected main bronchus with a bronchial occluder). A closed chest was established, and an artificial pneumothorax was maintained at a pressure of 4–6 mm Hg (1 mmHg = 0 133 kPa) at a flow rate of 2 L/min. Using the three-port method and 30° thoracoscopy, the observation port was located between the 5th intercostal line of the subscapular angle, and the two operating holes were located between the 7th intercostal line of the axillary midline and the 7th intercostal line between the erector spinae muscle and subscapular angle. Microscopic observations revealed that the right kidney had herniated into the chest cavity on the posterolateral side of the diaphragm. The right kidney was brought back into the abdominal cavity. The hernial sac was thin (Figure 2A). Upon incision of the hernia sac, a bright red lesion with clear boundaries and an abnormal blood vessel supply was observed (Figures 2B,C). After cutting off the abnormal blood vessels, LigaSure TM was used to remove the diseased tissue, and the renal fat sacs and renal tissue were visible (Figure 2D). Interrupted suturing of the hernia ring was performed to seal the diaphragmatic hernia. A retrieval bag was used to remove the diseased tissue, which was sent for examination. Upon confirming that there was no active bleeding or air leakage, and that the lungs were in good condition, a closed chest drainage tube was placed. The time of resecting the sac and reconstructing the diaphragm was 55 min.

**Figure 3:**
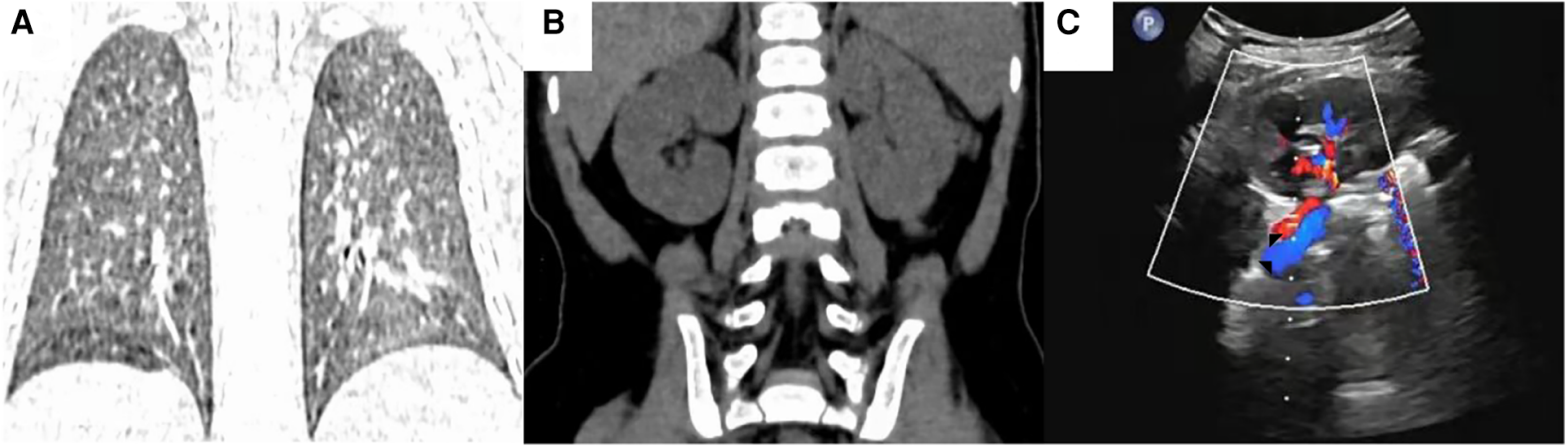
Follow up chest computed tomography (CT) imaging and renal ultrasound findings at 2 months post-surgery. **(A)** The chest CT coronal image showed that right diaphragm was in the normal position. **(B)** The chest CT coronal image showed that right kidney had returned to the abdominal cavity. **(C)** The renal ultrasound showed that blood flows in the right renal artery and vein were smooth. (◤: Right kidney artery, ◥: Right kidney vein).

Postoperative pathological examination revealed infradiaphragmatic pulmonary sequestration. The postoperative vital signs of the patient were stable. On postoperative day 3, the closed thoracic drainage tube was removed and the patient was discharged. Two months after surgery, chest CT showed that the right kidney had returned to the abdominal cavity and the right diaphragm was in a normal position, renal ultrasound showed that blood flow in the right renal artery and vein were smooth (Figure 3).

## 3 Discussion

Congenital PS is an independent, nonfunctional lung parenchyma that is not connected to the tracheobronchial tree and has an abnormal systemic arterial blood supply, which is usually from the thoracic or abdominal aorta (6). The infradiaphragmatic ELS is relatively rare. because of its location below the diaphragm and concealment, it is prone to misdiagnosis (7).

Infradiaphragmatic ELS is separate from normal lung tissue; therefore, the risk of infection is relatively low, but there is still a risk of malignant transformation or significant spontaneous bleeding (8, 9). Located between the kidney and diaphragm, it is difficult to distinguish infradiaphragmatic ELS from neuroblastoma, pheochromocytoma, and teratoma (10, 11). Therefore, early surgical resection should be performed in asymptomatic infradiaphragmatic ELS. Minimally invasive surgery has achieved good results for the treatment of PS. Because infradiaphragmatic ELS is located in the abdominal cavity, laparoscopic surgery is a better choice than thoracoscopic surgery (12, 13).

An ITK is defined as a kidney passing through a defect in the diaphragm into the thorax, but not into the pleural cavity. It may rotate or have a unipolar deviation and long ureter (14). ITK is usually located on the left side, accounting for 62% of cases, with the right side accounting for 36%. It is lower on the right side because the right pleural peritoneal channel closes earlier and is obstructed by the liver, which acts as a physical barrier (4, 5). ITK can be classified into four groups: (1) pure ITK with an intact diaphragm, (2) ITK with eventration of the diaphragm, (3) ITK with a congenital diaphragmatic hernia (Bochdalek's), and (4) ITK with a traumatic diaphragmatic hernia (15). ITK associated with CDH is extremely rare.

ITK associated with CDH usually requires surgical treatment because of the risk of respiratory distress and renal vascular torsion (16). With the development of minimally invasive technology, minimally invasive surgery can achieve the same surgical effect as open surgery, and has the advantages of minimal trauma, fast recovery, and fewer postoperative complications (5). Because this disease involves both the chest and abdominal cavity, it is more appropriate to choose thoracoscopic or laparoscopic surgery. Through a literature review, we found that although there are anatomical abnormalities in ITK associated with CDH, there are no complications such as hypertension, renal dysfunction, and proteinuria. Therefore, nephropexy is not necessary (4). For ITK associated with CDH, most scholars choose to close the diaphragmatic hernia without handling the child; therefore, simply performing CDH repair with thoracoscopic surgery is better than with laparoscopic surgery (5).

In the present case, the patient had infradiaphragmatic pulmonary sequestration and ITK associated with CDH. Therefore, there is confusion regarding the treatment choice. Is thoracoscopic surgery more appropriate than laparoscopic surgery? We found, through careful reading of the enhanced CT of the patient, the following: (1) The lesions in the patient were all located on the right side with liver obstruction, and the operating space under the diaphragm was small. Therefore, it was difficult to perform diaphragmatic hernia repair or infradiaphragmatic ELS resection through laparoscopy, whereas thoracoscopic surgery is relatively easy. (2) Although infradiaphragmatic ELS is located in the abdominal cavity, it protrudes from the upper wall of the hernial sac into the chest cavity and has a small abnormal blood supply. Through thoracoscopic surgery, the sequestrated lung can be identified by opening the hernial sac. The small abnormal supply of blood vessels can be cut off using the LigaSure TM, and the possibility of blood vessel rupture and bleeding is very low. Based on the above two analyses, thoracoscopic treatment was undoubtedly a better choice for this patient. We followed the preoperative surgical plan and opened the hernia sac during surgery. We found a bright red lesion with clear boundaries and an abnormal blood vessel supply. After cutting off the abnormal blood vessels, LigaSure TM was used to remove the lesion and the diaphragmatic hernia was repaired. The patient recovered smoothly after the surgery. Two months after surgery, chest CT showed that the right kidney had returned to the abdominal cavity, and the right diaphragm was in the normal position.

## 4 Conclusion

Infradiaphragmatic pulmonary sequestration and ITK associated with CDH is extremely rare. It can be diagnosed, and appropriate surgical plans can be developed, using enhanced CT. For infradiaphragmatic pulmonary sequestration located at the top of the hernia sac in CDH, thoracoscopic resection of the infradiaphragmatic pulmonary sequestration and repair of the diaphragmatic hernia is feasible and effective.

## Data Availability

The original contributions presented in the study are included in the article/Supplementary Material, further inquiries can be directed to the corresponding authors.
